# “When I woke up I was so worried and ashamed, I thought it was a disease”: Adolescent boys’ transitions through puberty in Kenya

**DOI:** 10.3389/frph.2022.956060

**Published:** 2022-11-07

**Authors:** Allison Carney, Titus Mulei, Daniel Kurao, Christine Hagstrom, Marni Sommer

**Affiliations:** ^1^ Independent Consultant, Nairobi, Kenya; ^2^ Amref Health Africa, Nairobi, Kenya; ^3^ Department of Population and Family Health, Columbia University Mailman School of Public Health, New York, NY, United States; ^4^ Department of Sociomedical Sciences, Columbia University Mailman School of Public Health, New York, NY, United States

**Keywords:** adolescents, puberty, maturation, boys, gender, health, Kenya

## Abstract

Growing evidence suggests a need for more focused attention on boys’ experiences of puberty in sub-Saharan Africa to assure healthy transitions into young adulthood. Existing research remains limited on the masculinity norms shaping boys’ maturation experiences in Kenya. To help fill this gap, we conducted a comparative case study using qualitative methodologies with 16–19-year-old male youth in rural and urban Kenya, and with adults interacting in boys’ daily lives. Findings suggest that Kenyan boys experience shame, confusion and silence around changes happening in their bodies; face pressures from new societal expectations as they become young men; and have adolescent lives shaped by minimal supervision, increased peer pressures and engagement in more risky health behaviors. Additional research and targeted interventions on boys transitioning through puberty and early adolescence are needed to better understand their vulnerabilities and prevent or reduce their engagement in unsafe behaviors.

## Introduction

In recent decades, health and education interventions in low- and middle-income countries have primarily focused on adolescent girls and young women (1, 2). This population is particularly vulnerable to sexual and gender-based violence, infection with HIV and AIDS, and gendered norms that reduce their educational and other opportunities (3, 4). Although the impact of the COVID-19 pandemic may be reversing hard-won gains, increased numbers of girls are enrolled in education; and in some countries, there are reduced rates of child marriage, early pregnancy, and infection with HIV and AIDS (5–7).

While young women's health and education are critical, there is increasing concern about the challenges that boys encounter during adolescence (8, 9). Although fewer girls than boys enroll in school in sub-Saharan Africa and South Asia, boys are less likely to progress or complete their education in particular regions, including Northern and Western Africa (10). Many boys leave school due to poor academic performance or family's inabilities to cover school-related expenses (10, 11). Gendered norms in many sub-Saharan African countries may also put pressure on young men to leave school and become providers for their families (10).

Adolescent boys and young men growing up in countries across sub-Saharan Africa also face multiple health-related vulnerabilities. In many contexts, as their bodies mature, they engage in employment or domestic chores requiring heavy labor, which may increase their risk for injuries (12–14). Some begin to use alcohol or other substances to cope with physically demanding jobs, as seen in studies in Kenya, Ghana, Uganda, and Tanzania (15–17). In South Africa, a study with young men and women aged 13–21 indicated boys become more susceptible to peer and societal influence during adolescence (18). A global systematic review further details that, across cultural settings, interpersonal and community factors influence stereotypical gender attitudes among young adolescents (19). Masculinity norms (e.g., emotional stoicism, physical toughness) encourage boys to take up various negative health behaviors, including substance use, unprotected sexual activity and violence with other boys and girls (20).

There is growing recognition of the importance of also intervening with boys and young men, both for their own well-being and to improve the health of girls and young women who are impacted by their behavior (21–23). This includes engaging boys in initiatives to reduce gender-based violence (24–26) or including young men in family planning (27). Findings from the Gender and Adolescence: Global Evidence (GAGE) study conducted in 34 low- and middle-income countries suggest that in some contexts, boys and men are seen as needing less guidance and support, given their perceived advantage for employment and increased autonomy as compared to girls and women (28). However, research from Tanzania and South Africa reinforces the problematic nature of this perspective, indicating how a lack of attention and guidance for maturing boys may increase their vulnerability to masculinity norms encouraging risky health behaviors (29, 30).

In Kenya, 85% of boys are enrolled in primary school, with differences in enrollment by region: 60% of boys in the Northeast region are enrolled in primary school as compared to 94% in the Central region (31). Educational attainment affects health outcomes and influences engagement in risky behavior (9, 32). In Kenya, boys who have not completed secondary education have less knowledge about preventing HIV than those completing school (31). Kenyan boys without a secondary education are also more likely to use tobacco, with 21% of boys who completed primary versus 10% of boys who completed secondary education reporting regular smoking (31). A study in Kilifi County found that boys who dropped out of school were more likely to engage in alcohol, drug use and theft (33).

Globally, the transition into young adulthood intensifies gendered norms (19, 34), bringing new pressures on maturing boys to demonstrate their manhood, including engaging in substance use, violence or initiating sexual relations (35). A study conducted in Eastern Cape, South Africa supported this finding, with researchers documenting how boys’ concepts of manhood were intensified through ritualistic practices, such as circumcision, and that much of the information boys learn about maturation occurs through such rituals and from pornography. This in turn was suggested to contribute to problematic beliefs around sex, masculinity, and relationships. The authors argue more robust, early, and formalized sexual and reproductive health curriculum could reduce future violence again women and girls resulting from masculinity norms (36).

Parent-child communication barriers may also hinder the conveyance of guidance to boys who are maturing in many contexts. A review of such communication dynamics in four countries across East Africa suggested challenges are bi-directional; boys are uncertain about who they can reach out to for advice, e.g., topics may feel taboo, while parents’ education levels or absence from home due to income generating activities, hinders their engagement. The study findings suggest that as a result adolescent boys may have insufficient guidance to navigate their new body changes, emotions, and societal pressures or expectations (37). In rural and urban Uganda, a study conducted with 10–14 year old boys and girls found that rural boys in particular often lacked adequate formal pubertal education and guidance; this in turn led many to turn to peers for information rather than potentially more credible adult sources (38). Early adolescence is an important window for addressing the patterns of gender socialization and understanding the information and guidance that boys receive during puberty is essential for designing interventions that meet their needs as they mature into young men.

## Methods

To contribute to the limited evidence on boys’ transitions through puberty in sub-Saharan Africa, we conducted a qualitative study with adolescent boys ages 16–19 in Kenya. Aiming to understand perspectives on puberty and becoming a young man in society, we, one, compared boys’ experiences of puberty changes, including personal understandings of masculinity and newer influences (internet, media); two, observed the influence of masculinity norms conveyed through family, peers and community on risky behaviors; and three captured recommendations on the guidance and support needed by boys transitioning into adulthood.

We applied an adapted version of Bronfenbrenner's socio-ecological model (39) to explore Kenyan boys’ puberty transitions. In particular, the study focused on the macro (e.g., how societal and gender norms shape masculinity), meso (e.g., what, and how, boys learn about puberty and development), and micro (e.g., boys’ personal experiences of puberty) levels of influence. The conceptual framework allowed an exploration of boys’ emotional and physical experiences, reflecting on their learning and relationships with their families and peers, school environment, community, and norms molding their perceptions of masculinity.

### Research setting

We conducted the study in rural and urban Kenya. The rural site was located along the coast of the Indian Ocean in Kwale County, which has a population of 1.7 million (40). Comprised of mostly nine coastal tribes, Kwale is predominately Muslim, with farming or livestock herding as the primary source of income. The urban location was Naivasha, Nakuru County, which has a population of 253,224 (41). A demographically diverse town, Naivasha is an urban center, with industries ranging from family farming to industrialized flower cultivation.

### Study design, instruments and sampling

We conducted a comparative case study (urban versus rural) from May to June in 2019 using multiple qualitative methodologies. A non-Kenyan woman (A.C.) and a young Kenyan man (T.M.), aged 24, spent two months conducting data collection. The non-Kenyan woman guided the interview process while the young Kenyan man asked the interview questions in Swahili and provided translation for the response. The principle investigator (M.S.) provided oversight daily through electronic communication.

### Data collection

#### Key informant interviews

We conducted key informant interviews with adults (*n* = 15) who shape adolescent boys’ environment, including health care workers, parents, teachers, police officers, religious leaders, and policymakers. A semi-structured interview guide included questions about masculinity norms in the community, rites of passages for boys, and the school environment for adolescents. Each interview lasted approximately 1.5 h and was held in a private room of the participant's choosing.

#### Focus group discussions

We conducted focus group discussions (FGDs) at primary schools (*n* = 6) in private classrooms that lasted approximately one hour. All FGDs were comprised of male and female teachers, mostly upper primary instructors (class 4 to class 8) and some lower primary instructors (class 1 to class 3). The FGD guide explored teachers’ observations of boys entering early adolescence, their insights on the curriculum, channels of information to boys, and school improvements that could better support boys. Primary school teachers were intentionally sampled given their close observations of boys undergoing the changes of puberty, including the social dynamics that occur around them. The boys themselves may not yet feel comfortable or able to discuss the pubertal changes that are occurring. This enabled a triangulation with the data emerging from the participatory activities with older adolescent boys described below.

#### Participatory activities

We conducted participatory activities with groups of in-school adolescent boys aged 16–19, stratified by class (Form 2–4), and out-of-school boys (each group up to 20 boys; 4 groups per site; 8 groups total; *n* = 130) (see Table 1). In-school participants were drawn from a government secondary school in each site, while out-of-school participants were recruited from vocational training centers. Older adolescent boys were intentionally sampled for the participatory activities as they have experienced puberty and are able to reflect on the physical, emotional, and social changes that occur along with providing recommendations for what boys growing up in Kenya today might need in terms of support and education.

**Table 1:** Sample characteristics of young men (aged 16–19) in Kenya.

Education status	Urban	Rural
Students	60	47
Out of school	11	12

Participatory methods included a range of anonymous and group activities (see Table 2) and were conducted in a private classroom at schools and vocational training centers. Each group met with the research team once weekly for three consecutive weeks. All participants provided informed consent. Given the sensitivity of the topics, group discussions were not recorded to ensure participants would feel comfortable and encourage participation. Careful, in-depth notetaking captured verbal responses and non-verbal behaviors. Written data, including stories and questions, were collected from young men anonymously. Each session lasted approximately 1.5 h.

**Table 2:** Participatory methods used in the study.

Study component	Method
*Written activities*	
Story writing	Participants were asked to write anonymous stories about puberty (e.g., their first experience of a wet dream or erection), a time they experienced peer pressure (e.g., did something they didn't want to do, or later regretted), and a time they witnessed violence or participated in a violent act
Puberty questions	Participants submit anonymous questions about puberty and growing up that they wanted answered
*Small group/pair activities*	
Puberty activities	Participants worked in groups to identify issues related to the health, social and emotional aspects of puberty and body changes
Peer pressure activity	Participants worked in groups to identify peer pressure and other influences they experienced as they transition through adolescence
Violence activity	Participants worked in groups to identify issues related to violence they have witnessed or experienced in their families, communities and society
Good and bad of being a man activity	Participants worked in groups to identify their perceptions on the good and bad characteristics or outcomes of being a man in their local society; larger group discussion analyzed the lists and identified additional aspects
Curriculum activity	Participants worked in groups to generate recommendations for a puberty curricula for youth in schools in Kenya

The activities were conducted in Kiswahili and English. Kiswahili data were translated for analysis by the larger research team.

### Analysis

We conducted thematic analysis of the transcripts from key informant interviews, FGDs and the participatory activities, and analyzed the adolescent boys’ written anonymous stories. Two members of the research team read through the data separately and generated themes together. Coding of the data was then conducted as a team, talking through analytic disagreements and confusion. We used open coding to classify categories, themes, and issues that emerged from the data.

The analysis identified three themes: (1) Shame, confusion, and silence; (2) Changing expectations as boys become young men; (3) Minimal supervision, peer pressure and risky behaviors; and (4) Gendered dynamics of puberty education. The thematic analysis did not reveal significant differences between the rural and urban sites selected for this study, however those that did emerge are highlighted throughout the four themes.

## Results

### Shame, confusion, and silence

Despite the cultural, religious, and economic differences of the rural and urban sites, adolescent boys in both locations reported feelings of guilt, shame, and confusion as their bodies changed. When asked about the onset of puberty, many boys expressed feeling uncertain and afraid, particularly after their first wet dream. One rural student explained: “*I felt so strange and scared…I was full of stress. I was thinking [about] what to do*.” Many boys thought they had urinated in their sleep, something that one is supposed to grow out of in early childhood. Others thought they were sick, as one boy in the urban site described feeling after his first wet dream:

…*I was amazed because I remembered that the last time I made my bed wet, I was in class 2, eight years old, and now I was in class 6 and it was shameful…. In my mind, I thought I was maybe sick or maybe slept and something went wrong*.

Many boys also shared feelings of loneliness and a sense of isolation, wondering if these body changes were only happening to them. The boy above went on to describe keeping his experience to himself, not sharing his concern with others. This perceived need to keep silent was reported by most boys.

The timing of puberty education may also contribute to boys’ confusion. Several of the primary teachers reported that although puberty and reproductive health topics are taught in upper primary, which is around when many boys are starting to experience body changes, timing is sometimes too late for those boys who have already started puberty. They further described how, particularly in rural areas, some boys might start school later than their peers, thus being further along developmentally by the time they receive formal education on the topic. Teachers and boys indicated that the curriculum should include more practical skills on managing or adjusting to body changes during puberty, in addition to basic biological and reproductive information. As another rural boy explained:

*Boys should learn about wet dreams, like when they appear, how you should manage them—class 6 and 7. We would tell them that the first time it happened, don’t panic, and don’t be shy, and don’t be surprised. They should wash their bodies and clean themselves with soap*.

Teachers and boys in both the urban and rural sites agreed that providing comprehensive puberty education prior to the onset of puberty would alleviate a lot of the boys’ confusion and fear.

Numerous adolescent boys described how they had experienced feelings of shame and embarrassment as their bodies changed, attributing a lack of information about what to expect as a contributing factor. Fear of being ridiculed worried many boys, such as when they experienced erections. Feelings of embarrassment extended to other pubertal changes, potentially impacting their academic performance and engagement. One urban teacher described observing how male students are embarrassed to verbally participate due to their cracking voice. During the participatory sessions, several adolescent boys in both study sites showed discomfort discussing these issues, and rather than say the word “erection,” used hand gestures.

Numerous boys also hesitated to tell anyone about their experiences with erections or wet dreams. Some boys described thinking these were penis diseases, with a culture of silence observed around sexual topics that impeded their ability to feel reassured. Many adolescent boys felt like they had done something wrong to trigger the change in their bodies, and it was thus inappropriate to discuss these changes with their parents. As one urban primary school teacher reported:

*It is the African Culture, so it is hard to talk openly. The way in which they are brought up, it is hard for boys to talk about issues openly. It is hard for parents to talk about the same because of culture and religion really limit these things*.

This finding was confirmed across both sites in the boys’ personal stories about their first wet dream or erection, where about half of the participants reported they did not tell anyone, at least for some time.

Of those who did describe seeking advice, most went to an older brother or peer rather than parents. Teachers reported that parents typically did not initiate conversations about puberty with boys, mostly due to social norms about what is discussed between parents and sons, but also because they felt unable to answer questions that young people might pose.

Structural challenges also affect parent-child communication, with several teachers describing how parents are often too busy working outside of the home to be present enough for these sensitive conversations. As one urban teacher explained: “*Children are more open to their teachers than their parents because the parents seem too busy. The parents are away most of the time, so students spend more time with their teachers than they do with their parents*.” Teachers in both study sites suggested that the lack of conversations at home, coupled with inadequate formal education that often comes too late, may leave many adolescent boys with insufficient knowledge and management skills, both emotionally and physically.

### Changing expectations as boys become young men

The signs of pubertal body changes were perceived by both boys and community members as a strong societal indicator that a boy was entering adulthood. A rural Community Health Promotion Officer explained: “*The development of the muscles, of the deep voice…of the beard…of pubic hair, this tells him now, he is a man*.” Several boys, particularly those no longer in school, reported feeling like their first wet dream or erection had been signs that they were no longer a child and had become grown men. Several key informants confirmed this societal perception of young adulthood, noting that there is an increased assertiveness and desire to be taken seriously after the onset of puberty amongst boys, along with potentially being seen as less easy to manage by some adults as they exert a newfound sense of independence. As a local urban Chief explained:

*[During puberty] some boys become unruly, they want to do their own things and get their freedom and be noticed that they are growing up…most want to be appreciated and want to be approved. Now I am man enough. They feel that they are equal to parents…*

One source of adolescent boys’ interpretation of signs and meanings of manhood may come from the tradition of circumcision that occurs in some Kenyan ethnic groups, like many boys from the urban location, after the onset of puberty. Among ethnic groups that practice circumcision, one becomes a man through being circumcised. A key aspect of the circumcision ceremony is for elders to educate boys about what it means to be a man and how they should behave within society. Some boys shared that it is also common to be introduced to drugs and sex during this transitional period, which was interpreted as indicating another aspect of societal masculinity norms being imparted to them about their expected behavior. As an urban nurse explained, society teaches boys that engaging in risk-taking is part of manhood:

*The community has expectations, no matter what you are taught. To be a real man, they have to smoke, drink, and have sex, and all of this starts after circumcision. The health care workers guide them, but they hear from the other boys and their peers and take up those behaviors…*

The nurse—along with other health workers and community members—described how although they seek to educate boys about the consequences of drugs and early sexual initiation, boys often resist their guidance because engaging in risky behaviors represents to them entering manhood.

Gender norms and family expectations were also described as influencing boys’ perceptions of what it means to be men. This includes the perception of men as the head of the household, with the economic and social expectations of providing financially for their families. Boys also described the importance of a man's role in defending his family and community, including using violence if necessary. As a rural teacher explained, being perceived as weak is taboo for men:

*A man is a tough person, very tough. Tough in the sense that he makes decisions that are not questionable, solidly takes care of the family, and does not cry in public. Maybe from class one, they are told men and boys don’t cry. [If he cries in public] he is ridiculed and becomes a laughing stock…*

Many participants emphasized the importance of men not crying in public because it would damage their reputation in the community. Part of showing strength as a man is not needing guidance from adults, including about body changes. An urban teacher explained: “*It [puberty information] is not something boys come to teachers for guidance on often. They feel like they are grown and do not need the help*.” Teachers also described that because boys are seen as men by their families, they often push back against school rules because they want to be seen as adults. Misbehavior and resistance to school rules were described as challenges by many teachers after boys reached puberty.

### Gendered dynamics of puberty education

Both boys and teachers described how boys receive minimal formal puberty education as their bodies matured, especially compared to female peers. Adolescent girls are reportedly provided extra lessons at school regarding sexual health and body changes, which boys described wanting as well. Beyond just his own body changes, a rural student described his desire to learn about the developmental changes that happen for boys and girls: “*Girls are taken somewhere and taught these things…we want to be included. Instead, it should be taught together. We will be fathers one day, so it is good for us to know. We should know that girls need [pads]*.”

Participants expressed gendered resentment of the girl-focused programming in schools. Several boys described feeling left out and frustrated when girls received menstrual materials from their schools or government. An urban boy explained: “*The girls get pads and panties at school, and we just watch like ‘Aye…’ There is too much for girls, so we feel very isolated…*” Most of the teachers agreed that boys would benefit from being provided with supportive materials during puberty education. When asked what should be done for boys, a rural teacher expressed:

*It should be all-inclusive. Not just the girl child alone. The boys should come together with the girls. When girls are given sanitary towels, the boys should be given boxers…. The boys in class, even when teaching and we say ok now it is time for the girls, they say, ‘what about us?’”*

While teachers acknowledged the increased vulnerability of girls and the need to focus on their education, safety, and well-being, they also reported that boys in Kenya had been left behind to a detrimental extent. An urban teacher explained: “*There are no NGOs or leaders trying to help the boys, only the girl child. Little is ever done for the boys. For the boy child and men, someone should come to their rescue*.” Teachers, community members, and boys themselves thought boys should be given more support during their transition into manhood.

### Minimal supervision, peer pressure and risky behaviors

The insights from the adolescent boys suggested that many are navigating new and challenging aspects of adolescence, such as increased access to technology and pornography—often with less supervision from parents or adults in their lives. This was found to potentially problematically shape their understandings of sex and relationships, of drug and alcohol use, or various forms of violence within society. After the onset of puberty, community members noted that it is typical for a boy to have increased freedom, including living in a separate house from his parents and spending less time with his family. An urban Senior Police Officer described how decreased parental supervision, a common cultural approach within local society, often results in boys engaging in risk-taking behaviors with older adolescents in the community: “*With their own homes, boys are told, ‘now you are a man’ and you are given a house of your own within the same compound. This can be manipulated by the older boys and they can fall into bad company*.” Older adolescent boys or young men may pressure younger boys to engage in sexual activity. In ethnic groups that undergo adolescent circumcision (typically around age 13, rather than at birth), which was the experience of most of the participating boys in the urban study location, boys are often pressured to have sex with girls as part of their initiation into manhood. As one adolescent health specialist from an urban NGO explained, the societal pressure to engage in sex can be quite intense:

*In this community, people do not talk openly [about body changes], so boys are left on their own and get information from their peers and it is not all correct. After their circumcision, they are told they have to sleep with a girl when they are healed, to signify that they have become a man. The boys believe that to be a real man, you need a number of girls, and you need to sleep with them…*

Several boys described the pressure from peers and older men to be in a relationship or have sex with a girl to fit in with their peers. Teachers noted that the pressure to have a girlfriend often leads boys to sexually harass girls and make them uncomfortable. Teachers and community members also perceived accessing pornography on cell phones to negatively influence boys and encourage early sexual initiation. A rural police officer expressed concern about the increased access to technology of boys growing up today: “*Masculinity was overtaken by technology…most of them can access mobile phones of parents and friends, they look at porn, so that triggers them to explore [in person].”* Together, the lack of parental supervision, peer pressure, and access to pornography were all perceived to influence boys’ early sexual initiation after reaching puberty.

In both sites, peer pressure was similarly described as problematic in influencing boys’ experimentation with alcohol and drugs. Many of the boys explained that they were often hesitant to try drugs but did so to be accepted and seen as both brave and manly by peers. As one urban boy described:

*…My fellow students had some drugs and they wanted to smoke. They gave me to try, and I refused, and they started laughing at me and saying I was not a man. I didn’t want to be accused, so I smoked the bangh (marijuana) for the first time…*

Pressure to do drugs seemed particularly common for younger boys, especially in the urban site, trying to fit in with older adolescents. Low-income boys often got involved with drugs, including selling them to make money to fit in with upper class boys. As an urban boy explained: “*When the changes come you feel like you are mature, and even if you’re 10 years, you walk with people who are maybe 25 years and maybe they do drugs, so then you start doing drugs and not respecting your parents, so then they don't pay your school fees, so you do more drugs…*” Teachers expressed concern that involvement with substance use led to poor academic performance, including dropping out of school entirely.

Boys and teachers also frequently mentioned increased violence and peer pressure to engage in fighting after the onset of puberty. Boys described how once they had started experiencing body changes, they felt grown and wanted to fight to feel like a man. A rural teacher described observing this behavior in school: “*The boys have temper tantrums and start being rude. There is a resistance now to the rules in school and to other boys. Like if they step on another boy, they do not apologize to show their strength of a man. They start bullying each other*.” Some community members expressed concern about how depictions of violence in video games and the media contributed to boys’ engaging in physical displays of strength with each other. Other adults suggested that the increased aggression observed among adolescent boys resulted from the natural hormonal changes, including increased testosterone, of puberty. Some key informants, ranging from teachers to police officers, described how adolescent boys were more likely to show increased physical aggression towards their parents. Some boys also mentioned that substance use contributed to their engagement in violence, as they did not think of future repercussions.

Overall, although many boys mentioned that seeking guidance from their parents is important if they experience peer pressure to engage in risky behaviors, most expressed discomfort, or unwillingness to ask their parents for advice. When asked why adolescent boys engage in risky behaviors, a rural teacher responded with a two-fold answer:

*First, there is a lack of knowledge. They don’t know the repercussions of their behavior. Two, they lack role models…People who are closer to their age and can show they have succeeded. If they have young mentors around them, not necessarily those who have only excelled in exams, but also other areas*.

The perspective of adults in Kenyan adolescent boys’ lives suggested that increased guidance on how to navigate the emotional and physical changes of puberty, through education and role models, would serve to reduce adolescent boys’ risk-taking behaviors, and lead to more positive health outcomes.

## Discussion

This study explored the pubertal experiences of adolescent boys in and out of school in one rural and one urban location in Kenya. We found that many boys experienced shame, confusion, and silence around the physical changes of puberty, with many also having felt unprepared for the ways in which their maturing bodies brought on new societal expectations. This included a reduction in adult supervision, which combined with intensifying gendered peer pressures, contributed to many boys becoming engaged in more risky behaviors. Overall, we found few differences between those living in rural versus urban areas, other than religious/cultural timing and rituals associated with circumcision, or between those who were in and out of school.

The findings that adolescent boys experienced feelings of anxiety and confusion about their pubertal body changes, such as wet dreams and erections, are similar to findings from studies conducted with adolescent boys in Cambodia and Tanzania; in both countries a number of boys interpreted the natural signals of maturation as a form of disease (30, 42). The Global Early Adolescent Study (GEAS), which explored experiences of early adolescence among youth and parents in Kenya and Nigeria, identified the same concerns among boys, although some also expressed pride in their physical maturation (43). These studies capture the ongoing cultural shame and stigma surrounding sexuality and physical body changes in many contexts, which in turn contribute to silence around these topics. Examining young people's exposure to guidance in high-income country contexts highlights a similar gap in puberty knowledge and information. A review of Australia's national curriculum for children aged 5, 10, and 15, identified that a “negligible” amount of puberty information was provided in school (44). In the United States of America (USA), an estimated half of all USA primary schools do not require puberty education, with many schools also providing options for parents to remove children from puberty lessons, which are often embedded within broader sexual health education (45).

Kenyan boys’ description of less supervision over their lives during their transition into young adulthood is similar to insights from other contexts. The GEAS study findings from six countries (Egypt, USA, Belgium, Nigeria, Kenya, China) suggested that while parents or caregivers of girls worried more about their safety and vulnerability after they began maturing, boys were afforded more freedom of movement and less monitoring, sometimes leading to adverse risky behavior, such as engaging in violence and drug or alcohol use (46, 47). The current study found that once Kenyan adolescent boys entered puberty, their own expectations and expressions of manhood also shifted. Another study conducted in Nairobi, Kenya found that pubescent boys begin to model their behavior and masculine identity on older boys and men in their surrounding community (48). Our findings support this dynamic, suggesting that following certain societal rites of passage, such as circumcision, Kenyan boys described being perceived as adults within their communities and assumed more freedom. This included many boys no longer living with parents or guardians, resulting in more opportunities for high-risk activities such as drinking alcohol or unsafe sex. A study conducted with adolescents aged 15 and older in urban Tanzania similarly identified reduced supervision of post-pubescent boys as a key factor increasing their vulnerability to engaging in the uptake of alcohol and other substances (49).

Insights from Kenyan boys in both the urban and rural sites describe an intensification of peer pressure and repercussions that create health vulnerabilities. This aligns with findings about boys from other parts of the world. Studies have found that adolescent boys and young men who do not feel support or that feel a sense of alienation are more likely to join peer groups or gangs in search of a sense of community; which may increase exposure to potentially negative influences (50, 51). In addition, studies conducted in Turkey, Trinidad and Tobago, the Caribbean, El Salvador, China, and Brazil showed that lower school attainment or violence experienced at school contributed to male gang involvement (52). In the absence of school or family structure, strong masculinity norms create a sense of belonging for boys, potentially resulting in adolescents choosing gangs or friend groups over school. In Kenya, programs have been introduced to address boys leaving school; however, most of them have focused on sexual and reproductive health rather than feelings of isolation and depression (53, 54), or hopelessness, which was expressed by some boys in the study discussed in this paper.

Finally, many Kenyan boys in this study were concerned with being perceived as “unmanly” if they asked older men information about confusing pubertal changes. Subsequently, many boys were left on their own and thus frequently sought (often incorrect) advice from older boys. This is similar to findings from Tanzania (30) and Cambodia (42), both of which revealed the silence around body changes experienced and maintained by boys and the older men in their lives, along with discomfort felt by parents to engage with puberty topics. In response to this challenge, several countries have begun approaches to engage boys in puberty education with positive outcomes. For example, in Iran, researchers found that using multimedia material on puberty content specifically for boys increased self-esteem (55). For example, a study conducted with secondary school male students in Pakistan recommended integrating puberty information for boys into a class module after discovering that boys lacked information and carried many misconceptions about their bodies, such as wet dreams being a sign of disease (56). Others have recommended effective ways to engage boys in comprehensive sexuality education (57). However much of the existing puberty education content, although limited in reach, continues to focus on girl's issues, such as menstrual health and hygiene, and not being adequately inclusive of boys’ pubertal changes (58).

While Kenya is making advancements in the implementation and scale of life skills education, which includes puberty content, delivery across the country is not uniform. Such information is not included on national exams, a positive approach given that inclusion as a testable subject might lead young people to find the content unappealing; however this also means that life skills education and sexual health content often gets overlooked by teachers (59, 60). In addition, the target age or grade level can pose challenges for young people. A UNESCO review of ten East and Southern African countries found that puberty content is often presented in upper primary or early secondary school, an age group who have usually already entered puberty (61). Hence, similar to the findings from the study described here, the information is conveyed late for the intention of reducing young people's concerns and confusion around the physical and social changes of pubertal onset. Early sexual and reproductive health curriculum, focused on puberty, including physical, emotional, and social changes, is an opportunity to provide critical health information as an early intervention to reduce vulnerability to outcomes such as teenage pregnancy, HIV, and sexual and gender-based violence. In countries like Kenya, where the delivery of comprehensive sexuality education is complex, puberty content interventions might be a more effective approach for the education system and other youth-focused organizations to reach boys (62). Overall, the world has a long way to go with providing puberty education that address the needs of both girls and boys. However, the findings from this study reinforce the importance of engaging boys in puberty education in addition to current efforts supporting girls worldwide.

## Strengths and limitations

The use of participatory methodologies with boys is a useful approach for eliciting their insights and recommendations. Such approaches enable a more trusting and empowered exchange with participants. Although our findings provide deeper insights into the experiences of boys transitioning through puberty in rural and urban Kenya, given the qualitative study design and small sample of boys in particular regions and ethnic groups, the findings are not generalizable to all of Kenya.

## Conclusion

This study captured valuable insights into masculinity norms shaping Kenyan boys’ transitions into young adulthood and their own reflections and recommendations for how greater information and guidance might better support all boys. More extensive research is needed on each of the topical areas that were explored and arose from the participatory methodologies and interviews, including how better to support boys who are navigating peer pressures to engage in risky sexual behaviors, to uptake and use substances, and the modeling of violence around them.

## Data Availability

The datasets presented in this article are not readily available because the IRB did not include approval of the datasets. Requests to access the datasets should be directed to ms2778@columbia.edu.

## References

[1] Fantaye AW Buh AW Idriss-Wheeler D Fournier K Yaya S. Effective educational interventions for the promotion of sexual and reproductive health and rights for school-age children in low- and middle-income countries: a systematic review protocol. Syst Rev. (2020) 9(1):1–8. 10.1186/s13643-020-01464-w 32948251 PMC7500715

[2] Haberland NA McCarthy KJ Brady M. A systematic review of adolescent girl program implementation in low- and middle-income countries: evidence gaps and insights. J Adolesc Health. (2018) 63(1):18–31. 10.1016/j.jadohealth.2017.11.294 29434004

[3] https://www.avert.org/professionals/hiv-social-issues/key-affected-populations/women.

[4] Wodon Q Montenegro C Nguyen H Onagoruwa A. Missed opportunities: the high cost of not educating girls. World Bank (2018). Available at: https://openknowledge.worldbank.org/bitstream/handle/10986/29956/HighCostOfNotEducatingGirls.pdf?sequence=6&isAllowed=y

[5] McDougal L Shakya H Dehingia N Lapsansky C Conrad D Bhan N Mapping the patchwork: exploring the subnational heterogeneity of child marriage in India. SSM - Popul Health. (2020) 12:100688. 10.1016/j.ssmph.2020.100688 33319026 PMC7726340

[6] Somani T. Importance of educating girls for the overall development of society: a global perspective. J Educ Res Pract. (2017) 7(1):125–39. 10.5590/JERAP.2017.07.1.10

[7] Global Education Monitoring Report Team. Education for people and planet: creating sustainable futures for all: Global Education Monitoring Report 2016. Paris: UNESCO (2016).

[8] Gottert A Barrington C McNaughton-Reyes HL Maman S MacPhail C Lippman SA Gender norms, gender role conflict/stress and HIV risk behaviors among men in mpumalanga, South Africa. AIDS Behav. (2018) 22(6):1858–69. 10.1007/s10461-017-1706-9 28161801 PMC6440537

[9] Jewkes R Flood M Lang J. From work with men and boys to changes of social norms and reduction of inequities in gender relations: a conceptual shift in prevention of violence against women and girls. Lancet. (2015) 385(9977):1580–9. 10.1016/S0140-6736(14)61683-4 25467578

[10] Global Education Monitoring Report Team. Achieving gender equality in education: don’t forget the boys. UNESCO (2018).

[11] Burns M. Leaving boys behind? Global Partnership for Education (2019). Available at: https://www.globalpartnership.org/blog/leaving-boys-behind

[12] Ibrahim A Abdalla SM Jafer M Abdelgadir J de Vries N. Child labor and health: a systematic literature review of the impacts of child labor on child’s health in low- and middle-income countries. J Public Health. (2018) 41(1):18–26. 10.1093/pubmed/fdy018 PMC6459361 29409061

[13] Schlick C Joachin M Briceño L Moraga D Radon K. Occupational injuries among children and adolescents in Cusco province: a cross-sectional study. BMC Public Health. (2014) 14(1):1–8. 10.1186/1471-2458-14-766 25074757 PMC4122785

[14] WHO and UNICEF. World report on child injury prevention (2008). 26269872

[15] Boua PR Soo CC Debpuur C Maposa I Nkoana S Mohamed SF Prevalence and socio-demographic correlates of tobacco and alcohol use in four sub-saharan african countries: a cross-sectional study of middle-aged adults. BMC Public Health. (2020) 21(1):1–14. 10.1186/s12889-021-11084-1 PMC8196437 34118914

[16] Kuteesa MO Seeley J Cook S Webb EL. Multi-level experiences and determinants of alcohol misuse and illicit drug use among occupational groups at high-risk of HIV in sub-saharan Africa: a thematic synthesis of qualitative findings. Glob Public Health. (2019) 15(5):715–33. 10.1080/17441692.2019.1679216 31640453 PMC7175470

[17] Sommer M Kaaya S Kajula L Marwerwe G Hamisi H Parker R. Social and structural determinants of youth alcohol use in Tanzania: the role of gender, social vulnerability and stigma. Glob Public Health. (2020) 16(1):75–87. 10.1080/17441692.2020.1801792 32744916 PMC7790840

[18] Choe DE Zimmerman MA Devnarain B. Youth violence in South Africa: exposure, attitudes, and resilience in zulu adolescents. Violence Vict. (2012) 27(2):166–81. 10.1891/0886-6708.27.2.166 22594214

[19] Kågesten A Gibbs S Blum RW Moreau C Chandra-Mouli V Herbert A Understanding factors that shape gender attitudes in early adolescence globally: a mixed-methods systematic review. Plos One. (2016) 11(6):1–36. 10.1371/journal.pone.0157805 PMC4920358 27341206

[20] Amin A Kågesten A Adebayo E Chandra-Mouli V. Addressing gender socialization and masculinity norms among adolescent boys: policy and programmatic implications. J Adolesc Health. (2018) 62(3):S3–S5. 10.1016/j.jadohealth.2017.06.022 29455715 PMC5817048

[21] Gwyther K Swann R Casey K Purcell R Rice SM. Developing young men’s wellbeing through community and school-based programs: a systematic review. Plos One. (2019) 14(5):1–20. 10.1371/journal.pone.0216955 PMC6527294 31107908

[22] Ruane-McAteer E Hanratty J Lynn F Reid E Khosla R Amin A Protocol for a systematic review: interventions addressing men, masculinities and gender equality in sexual and reproductive health: an evidence and gap map and systematic review of reviews. Campbell Syst Rev. (2018) 14(1):1–24. 10.1002/CL2.203 PMC8428064 37131371

[23] Salam RA Das JK Lassi ZS Bhutta ZA. Adolescent health interventions: conclusions, evidence gaps, and research priorities. J Adolesc Health. (2016) 59(4):S88–92. 10.1016/j.jadohealth.2016.05.006 27664599 PMC5026678

[24] Crooks CV Jaffe P Dunlop C Kerry A Exner-Cortens D. Preventing gender-based violence among adolescents and young adults: lessons from 25 years of program development and evaluation. Violence Against Women. (2018) 25(1):29–55. 10.1177/1077801218815778 30803428

[25] Kågesten AE Oware PM Ntinyari W Langat N Mboya B Ekström AM. Young people’s experiences with an empowerment-based behavior change intervention to prevent sexual violence in Nairobi informal settlements: a qualitative study. Glob Health Sci Pract. (2021) 9(3):508–22. 10.9745/GHSP-D-21-00105 34593578 PMC8514032

[26] Miller E Jones KA Culyba AJ Paglisotti T Dwarakanath N Massof M Effect of a community-based gender norms program on sexual violence perpetration by adolescent boys and young men. JAMA Network Open. (2020) 3(12):e2028499. 10.1001/jamanetworkopen.2020.28499 33351083 PMC7756236

[27] Vouking MZ Evina CD Tadenfok CN. Male involvement in family planning decision making in sub-saharan Africa- what the evidence suggests. Pan Afr Med J. (2014) 19:1–5. 10.11604/pamj.2014.19.349.5090 25922638 PMC4406389

[28] Marcus R Stavropoulou M Archer-Gupta N. Programming with adolescent boys to promote gender-equitable masculinities: a rigorous review. Available at: https://www.gage.odi.org/wp-content/uploads/2018/12/Masculinities-Review-WEB1.pdf

[29] Selikow T-A Ahmed N Flisher AJ Mathews C Mukoma W. I am not “umqwayito'': a qualitative study of peer pressure and sexual risk behaviour among young adolescents in Cape Town, South Africa. Scand J Public Health. (2009) 37(2_suppl):107–12. 10.1177/1403494809103903 19493988

[30] Sommer M Likindikoko S Kaaya S. Parents, sons, and globalization in Tanzania. Boyhood Stud. (2013) 7(1):43–63. 10.3149/thy.0701.43 PMC8375493 34422152

[31] Kenya National Bureau of Statistics, National AIDS Control Council, Kenya Medical Research Institute, National Council for Population and Development, ICF International. Kenya demographic and health survey, 2014. Rockville, MD (2015).

[32] Keller J Mboya BO Sinclair J Githua OW Mulinge M Bergholz L A 6-week school curriculum improves boys’ attitudes and behaviors related to gender-based violence in Kenya. J Interpers Violence. (2016) 32(4):535–57. 10.1177/0886260515586367 26063788

[33] Ssewanyana D van Baar A Mwangala PN Newton CR Abubakar A. Inter-relatedness of underlying factors for injury and violence among adolescents in rural coastal Kenya: a qualitative study. Health Psychol Open. (2019) 6(1):1–11. 10.1177/2055102919849399 PMC6537266 31205735

[34] Hill JP Lynch ME. The intensification of gender-related role expectations during early adolescence. In: Brooks-Gunn J, Petersen AC, editors. Girls at puberty: Biological and psychosocial perspectives. Boston, MA: Springer. (1983). p. 201–28.

[35] Chandra-Mouli V Plesons M Amin A. Addressing harmful and unequal gender norms in early adolescence. Nat Hum Behav. (2018) 2(4):239–40. 10.1038/s41562-018-0318-3 30936534

[36] Hodes R Gittings L. ‘Kasi curriculum’: what young men learn and teach about sex in a South African township, sex education. Sex Educ. (2019) 19(4):436–54. 10.1080/14681811.2019.1606792

[37] Kamangu AA John MR Nyakoki SJ. Barriers to parent-child communication on sexual and reproductive health issues in east Africa: a review of qualitative research in four countries. J Afr Stud Dev. (2017) 9(4):45–50. 10.5897/JASD2016.0410

[38] Bunoti SN Tumwesigye NM Atuyambe L. Awareness of pubertal body changes among primary school children aged 10–14 years in eastern Uganda: challenges and opportunities. Reprod Health. (2022) 19(180):2–10. 10.1186/s12978-022-01466-y 35986331 PMC9392265

[39] Bronfenbrenner U. The ecology of human development. Cambridge, MA and London, England: Harvard University Press (1979).

[40] Kwale county background & history. Kwale county government. Available at: https://kwalecountygov.com/kwale/ (Accessed January 26, 2022).

[41] Nakuru country. Nakuru county government. Available at: https://nakuru.go.ke/ (Accessed January 26, 2022).

[42] Scandurra L Khorn D Charles T-A Sommer M. Cambodian boys’ transitions into young adulthood: exploring the influence of societal and masculinity norms on young men’s health. Cult Health Sex. (2016) 19(7):767–80. 10.1080/13691058.2016.1259504 27881047

[43] Bello BM Fatusi AO Adepoju OE Maina BW Kabiru CW Sommer M Adolescent and parental reactions to puberty in Nigeria and Kenya: a cross-cultural and intergenerational comparison. J Adolesc Health. (2017) 61(4):S35–41. 10.1016/j.jadohealth.2017.03.014 28915991

[44] Collier-Harris CA Goldman JD. Is puberty education evident in Australia’s first national curriculum? Sex Educ. (2016) 17(1):57–72. 10.1080/14681811.2016.1225259

[45] Sex and HIV education. Guttmacher Institute (2022). Available at: https://www.guttmacher.org/state-policy/explore/sex-and-hiv-education (Accessed January 26, 2022).

[46] Mmari K Moreau C Gibbs SE De Meyer S Michielsen K Kabiru CW ‘Yeah, i’ve grown; I can’t go out anymore’: differences in perceived risks between girls and boys entering adolescence. Cult Health Sex. (2017) 20(7):787–98. 10.1080/13691058.2017.1382718 29043890

[47] Woog V Kågesten A. The sexual and reproductive health needs of very young adolescents aged 10–14 in developing countries: what does the evidence show? Guttmacher Institute (2020). Available at: https://www.guttmacher.org/report/srh-needs-very-young-adolescents-in-developing-countries (Accessed January 26, 2022).

[48] Maina BW Sikweyiya Y Ferguson L Kabiru CW. Conceptualisations of masculinity and sexual development among boys and young men in korogocho slum in Kenya. Cult Health Sex. (2020) 24(2):226–40. 10.1080/13691058.2020.1829058 33289439

[49] Carney A Kaaya S Kajula L Ibitoye M Marwerwe G Sommer M. ‘Most of the youth are drinking because they have nothing to do’: how idle time facilitates adolescent alcohol use in urban Tanzania. J Child Adolesc Subst Abuse. (2020) 29(2):129–42. 10.1080/1067828X.2021.1888169

[50] AACAP. Gangs and children. American Academy of Child and Adolescent Psychiatry (2016). Available at: https://www.aacap.org/aacap/families_and_youth/facts_for_families/fff-guide/Children-and-Gangs-098.aspx

[51] Lenzi M Sharkey J Vieno A Mayworm A Dougherty D Nylund-Gibson K. Adolescent gang involvement: the role of individual, family, peer, and school factors in a multilevel perspective. Aggress Behav. (2014) 41(4):386–97. 10.1002/ab.21562 25288165

[52] Higginson A Benier K Shenderovich Y Bedford L Mazerolle L Murray J. Factors associated with youth gang membership in low- and middle-income countries: a systematic review. Campbell Syst Rev. (2018) 14(1):1–128. 10.4073/csr.2018.11 PMC8427992 37131383

[53] Hallfors DD Cho H Hartman S Mbai I Ouma CA Halpern CT. Process evaluation of a clinical trial to test school support as HIV prevention among orphaned adolescents in western Kenya. Prev Sci. (2017) 18(8):955–63. 10.1007/s11121-017-0827-8 28733854 PMC5693647

[54] Osborn TL Venturo-Conerly KE Wasil AR Schleider JL Weisz JR. Depression and anxiety symptoms, social support, and demographic factors among Kenyan high school students. J Child Fam Stud. (2019) 29(5):1432–43. 10.1007/s10826-019-01646-8

[55] Alimohammadi M Samani LN Khanjari S Haghani H. The effects of multimedia-based puberty health education on male students’ self- esteem in the middle school abstr. Int J Community Based Nurs Midwifery. (2019) 7(2):109–17. 10.30476/IJCBNM.2019.44882 31041321 PMC6456763

[56] Ul Huda S Mobeen K Idrees S Chagani P Zafar M. Knowledge of pubertal changes and self-care in adolescent boys. J Liaquat Univ Med Health Sci. (2017) 16(02):121–5. 10.22442/jlumhs.171620519

[57] Kato-Wallace J Barker G Sharafi L Mora L Lauro G. Adolescent boys and young men: engaging them as supporters of gender equality and health and understanding their vulnerabilities. UNFPA (2016).

[58] Crockett LJ Deardorff J Johnson M Irwin C Petersen AC. Puberty education in a global context: knowledge gaps, opportunities, and implications for policy. J Res Adolesc. (2019) 29(1):177–95. 10.1111/jora.12452 30869838

[59] Sidze EM Stillman M Keogh S Mulupi S Egesa CP Leong M From paper to practice: sexuality education policies and their implementation in Kenya. New York: Guttmacher Institute (2017). Available at: https://www.guttmacher.org/report/sexuality-education-kenya (Accessed September 25, 2022).

[60] Cynthia Khamala Wangamati. Comprehensive sexuality education in sub-saharan Africa: adaptation and implementation challenges in universal access for children and adolescents. Sex Reprod Health Matters. (2020) 28(2):1851346. 10.1080/26410397.2020.1851346 33295853 PMC7887764

[61] Birungi H Undie C MacKenzie I Katahoire A Obare F Machawira P. Education sector response to early and unintended pregnancy: a review of country experiences in Sub-Saharan Africa. STEP UP and UNESCO Research Report (2015). Available at: https://www.popcouncil.org/uploads/pdfs/2015STEPUP_EducSectorResp.pdf (Accessed September 24, 2022)

[62] Sommer M. An overlooked priority: puberty in sub-saharan Africa. Am J Public Health. (2011) 101(6):979–81. 10.2105/AJPH.2010.300092 21493937 PMC3093270

